# CNN-TumorNet: leveraging explainability in deep learning for precise brain tumor diagnosis on MRI images

**DOI:** 10.3389/fonc.2025.1554559

**Published:** 2025-03-26

**Authors:** Novsheena Rasool, Niyaz Ahmad Wani, Javaid Iqbal Bhat, Sandeep Saharan, Vishal Kumar Sharma, Bassma Saleh Alsulami, Hind Alsharif, Miltiadis D. Lytras

**Affiliations:** ^1^ Department of Computer Science, Islamic University of Science and Technology, Awantipora, Kashmir, India; ^2^ School of Computer Science and Engineering, Institute of Integrated Learning in Management University (IILM), Greater Noida, Uttar Pradesh, India; ^3^ School of Computer Science Engineering and Technology, Bennett University, Greater Noida, Uttar Pradesh, India; ^4^ Senior Project Engineer, AI Research Centre - Woxsen University, Hyderabad, Telangana, India; ^5^ Faculty of Computing and Information Technology, King Abdulaziz University, Jedda, Saudi Arabia; ^6^ Computer Science and Artificial Intelligence Department, College of Computing, Umm Al-Qura University, Makkah, Saudi Arabia; ^7^ Immersive Virtual Reality Research Group, King Abdulaziz University, Jeddah, Saudi Arabia; ^8^ Department of Computer Science and Engineering, American College of Greece, Athens, Greece

**Keywords:** brain tumor, MRI, classification, deep learning, explainability

## Abstract

**Introduction:**

The early identification of brain tumors is essential for optimal treatment and patient prognosis. Advancements in MRI technology have markedly enhanced tumor detection yet necessitate accurate classification for appropriate therapeutic approaches. This underscores the necessity for sophisticated diagnostic instruments that are precise and comprehensible to healthcare practitioners.

**Methods:**

Our research presents CNN-TumorNet, a convolutional neural network for categorizing MRI images into tumor and non-tumor categories. Although deep learning models exhibit great accuracy, their complexity frequently restricts clinical application due to inadequate interpretability. To address this, we employed the LIME technique, augmenting model transparency and offering explicit insights into its decision-making process.

**Results:**

CNN-TumorNet attained a 99% accuracy rate in differentiating tumors from non-tumor MRI scans, underscoring its reliability and efficacy as a diagnostic instrument. Incorporating LIME guarantees that the model’s judgments are comprehensible, enhancing its clinical adoption.

**Discussion:**

Despite the efficacy of CNN-TumorNet, the overarching challenge of deep learning interpretability persists. These models may function as ”black boxes,” complicating doctors’ ability to trust and accept them without comprehending their rationale. By integrating LIME, CNN-TumorNet achieves elevated accuracy alongside enhanced transparency, facilitating its application in clinical environments and improving patient care in neuro-oncology.

## 1 Introduction

Brain tumors are abnormal growths within the central nervous system that can significantly impact neurological function and overall health. The manifestations of brain tumors might differ markedly depending on their dimensions, position, and classification (1). Prevalent symptoms encompass persistent headaches, seizures, cognitive deficits, visual disturbances, and motor dysfunction. The intricate and vital processes of the brain render the diagnosis and treatment of brain tumors a significant issue. Malignant tumors are notably severe, signifying aggressive and life-threatening illnesses (2).Advanced imaging techniques, including computed tomography (CT), magnetic resonance imaging (MRI), and positron emission tomography (PET), are essential for the diagnosis and visualization of malignant tumors. MRI is widely recognized as the preferred modality for brain imaging due to its ability to provide detailed anatomical images without the risks associated with radiation exposure. It is particularly effective in identifying gliomas and accurately assessing their size, location, and relationship with surrounding tissues. Contrast-enhanced MRI further enhances the ability to distinguish malignant tumors from normal brain tissue. Gliomas are among the most common and aggressive forms of brain cancer, originating from glial cells that support neuronal functions in the brain and spinal cord (2). These tumors are heterogeneous and present significant challenges for accurate diagnosis, often requiring sophisticated classification methods. Traditionally, gliomas are classified into subgroups, such as astrocytomas, oligodendrogliomas, and ependymomas, based on the type of glial cells they originate from. High-grade gliomas, particularly glioblastoma multiforme (GBM), are characterized by aggressive growth and resistance to treatment, contributing to a poor prognosis (3). Despite advancements in treatment, high-grade gliomas remain a leading cause of mortality among patients with brain tumors (4). Early detection and precise categorization of gliomas are crucial for guiding treatment decisions and improving patient outcomes. However, the manual analysis of MRI images is time-consuming, prone to human error, and relies heavily on radiologists’ expertise. As a solution, there has been a growing shift toward automating this process using machine learning (ML) and deep learning (DL) techniques, which offer promising avenues for enhanced diagnostic accuracy (5–7).

### 1.1 Contribution

We introduce CNN-TumorNet, an innovative method for brain tumor classification utilizing a deep convolutional neural network (CNN) for the binary classification of MRI data. The model incorporates several CNN layers, batch normalization, max-pooling, and dropout techniques to enhance feature extraction. CNN-TumorNet accurately distinguishes between tumor and non-tumor brain tissue, achieving a classification accuracy of 99.9%. Recognizing that DL models are typically regarded as black-box systems, we enhance the interpretability of our CNN-TumorNet by incorporating the LIME (local interpretable model-agnostic explanations) technique. This *post-hoc* explainability approach provides insights into the model’s decision-making procedure, particularly for malignant glioma classification, enhancing transparency and building trust in its predictions.

## 2 Related work

**Figure 1:**
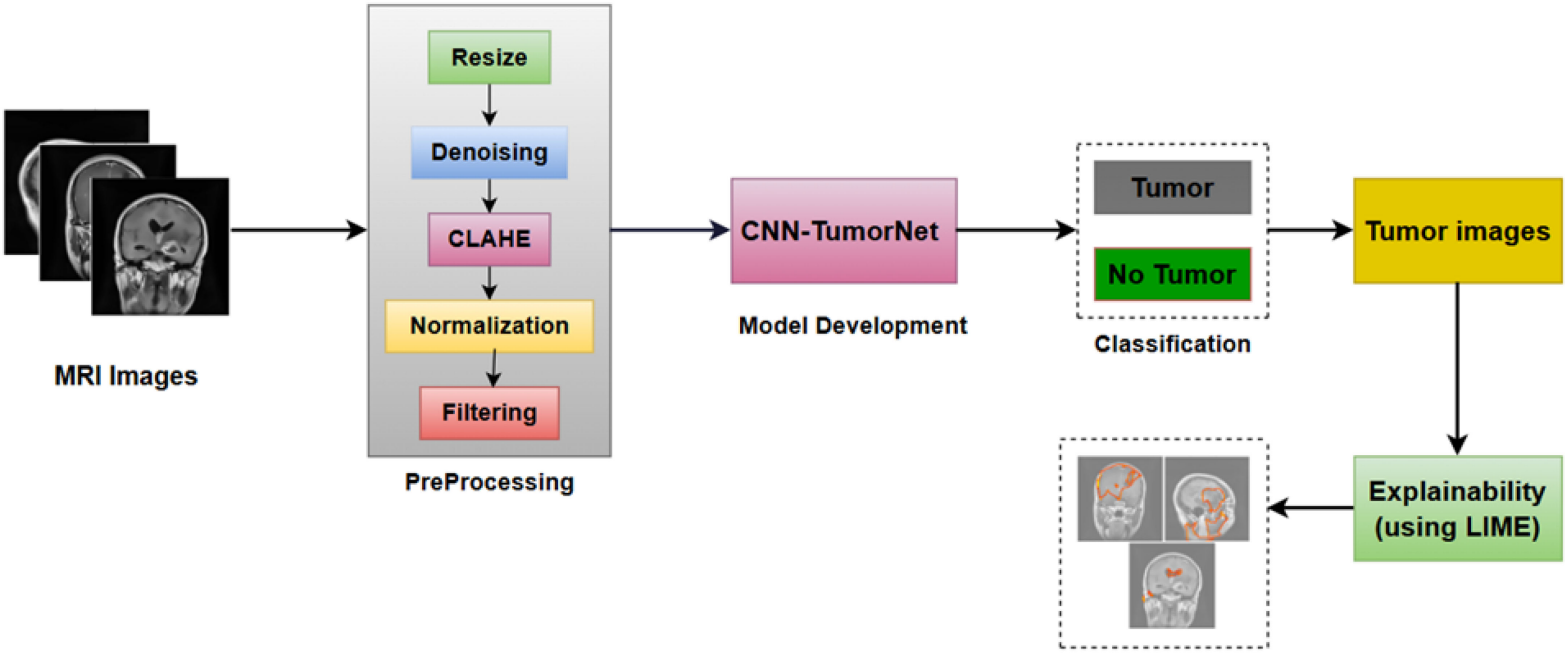
Workflow of the proposed method.

CNNs, in particular, have demonstrated remarkable efficacy in automatically detecting and classifying gliomas from MRI images. By training on large datasets of labeled MRI images, CNNs can identify subtle patterns and characteristics that distinguish gliomas from other brain tumors (8). Furthermore, CNNs can be fine-tuned to classify glioma subtypes based on distinct imaging features. Alternative machine learning approaches, such as Support Vector Machines (SVM), Random Forest (RF), and deep reinforcement learning (RL), have also been explored to improve glioma diagnosis and classification (9). Accurate tumor classification enhances diagnostic confidence, reduces patient anxiety, and aids in the selection of appropriate treatment plans (10). In addition, recent developments in Explainable Artificial Intelligence (XAI) aim to increase the transparency of these models, addressing concerns about their ‘black-box’ nature and making them more interpretable for clinical use (11).This work proposes a novel glioma classification model that integrates a sophisticated CNN architecture with XAI techniques to improve performance and interpretability. The model’s workflow, as illustrated in **Figure 1**, begins with the preprocessing of MRI images, ensuring that the data is adequately prepared for analysis. Next, the CNN-TumorNet classification network is trained and validated to distinguish between benign and malignant tumors. After classification, the LIME method is applied to elucidate the model’s decision-making process, enhancing the understanding of how it arrives at its predictions. This article is structured into six sections for clarity and comprehensive discussion: Section 1 presents the context and emphasizes the key contributions of this work. Section 2 delineates the approach, encompassing specifics regarding the dataset and the techniques for its preparation. Section 3 provides a comprehensive network architecture analysis, facilitating readers’ comprehension of the technological background. Section 4 addresses the explainability process, elucidating the interpretability of the model’s decisions. Section 5 delineates the conclusions derived from the study, while Section 6 encapsulates the principal findings and examines prospective avenues for further research. Tariq et al. (12) propose a lightweight human activity recognition method for video surveillance called SDIGRU, which integrates spatial and deep features extracted via MobileNetV2 to improve accuracy. The method uses a multilayer GRU to capture temporal dynamics in video frames while maintaining low computational complexity and fast response times. Experiments on benchmark datasets (HMDB51, YouTube11, UCF101) show that SDIGRU outperforms state-of-the-art techniques with superior recognition performance and efficiency. Tariq et al. (13) propose a human activity recognition method using CNN for feature extraction and Bi-GRU to capture temporal dynamics in video frames. By selecting key features, they reduce complexity and improve performance. Experiments on YouTube11, HMDB51, and UCF101 datasets show their method’s effectiveness compared to existing techniques. Yawar Abbas et al. (14) introduce the ECMT framework, combining memory analysis with ensemble machine learning to detect IoT malware. By integrating models like AdaBoost, ECMT achieves 96% accuracy in identifying malware families, such as ransomware and trojans. The framework is scalable, adaptable, and addresses concept drift, offering a robust solution for IoT cybersecurity. Asif Rehman et al. (15) developed a machine learning-based Intrusion Detection System (IDS) for identifying attacks and anomalies in Smart Home IoT environments. Using data from 41 IoT devices and 13 network traffic features, they preprocessed and stratified the dataset to build predictive models. They introduced a new Logit-Boosted algorithm, the Logi-CatBoost Classifier (Logi-CBC), which achieved the highest precision among similar algorithms with an accuracy of 88.70%. Their research highlights the effectiveness of Logi-CBC in classifying IoT device traffic and detecting abnormalities.

## 3 Methodology

### 3.1 Dataset description

**Figure 2:**
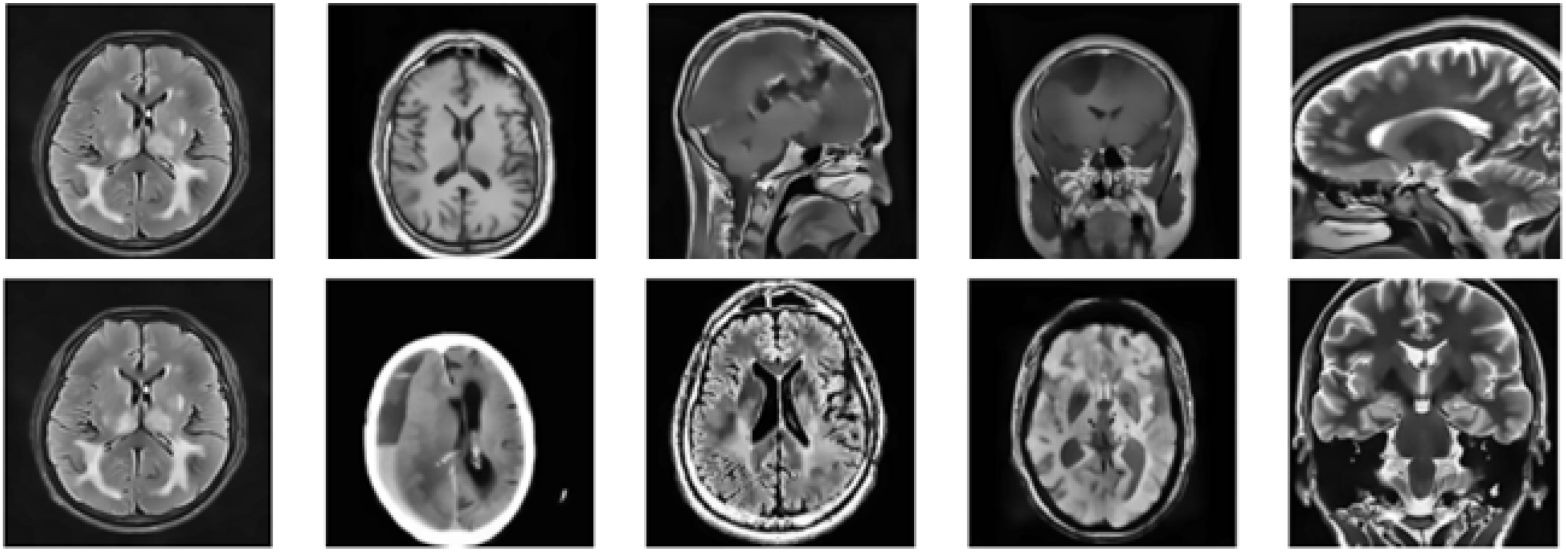
A few MRI images showing both benign and malignant cases.

The brain tumor MRI dataset utilized in our research includes 7,023 MRI images, rigorously separated into 4 specific categories such as glioma (300 MRIs), meningioma (306 MRIs), pituitary tumor (300 MRIs), and no tumor (405 MRIs) (16). These images are freely accessible in jpeg format, making them very suitable for ML applications, especially those focused on binary or multiclass classification jobs (see **Figure 2**). For our study, we emphasize the glioma and no tumor categories, reducing the dataset to meet our specific goal of binary classification. This approach enables us to effectively organize the dataset for our experimental objectives while maintaining a balanced representation of the two classes. Furthermore, we annotated the dataset to ensure consistency and accuracy. This stage confirms that the labels and data arrangement are wholly aligned with our study’s specific needs, increasing the reliability of the experimental results. The dataset does not provide details on the diversity of patient demographics, such as age, gender, and ethnicity. The class distribution in the dataset is as follows: 25.5% pituitary adenoma, 23.1% glioma, 23.4% meningioma, and 27.9% no tumor. This distribution suggests potential class imbalances that could introduce bias, affecting the model’s performance across different categories. Bibi et al. (17), used data augmentation to generalize the dataset.

### 3.2 Preprocessing

Preprocessing is crucial for the efficacy of machine learning models, particularly in the analysis of medical images (18). This phase entails standardizing input data to improve computational efficiency, enhance model accuracy, and facilitate interpretability. Below, we analyze how each preprocessing technique contributes to these objectives in the context of glioma identification and classification.

Downsizing Images: All MRI images, including those of gliomas and non-tumorous tissues, were resized to uniform dimensions to ensure compatibility with the neural network input requirements. This step reduces computational overhead and prevents inconsistencies caused by varying image resolutions, leading to improved convergence during training and better generalization.

Non-Local Means Denoising: The Non-Local Means Denoising technique was implemented to reduce noise while preserving essential image details, such as the intricate structures visible in MRI scans. This method enhances the signal-to-noise ratio, ensuring that the model focuses on diagnostically significant features rather than noise artifacts, thereby improving the accuracy and the interpretability of feature extraction by the model.

Contrast Limited Adaptive Histogram Equalization (CLAHE): CLAHE was employed to enhance image contrast, particularly in underexposed regions. This technique improves the visibility of subtle details in dimly illuminated areas of MRI images, which is critical for detecting fine tumor boundaries and textures. By amplifying these critical details without over-enhancing noise, CLAHE directly contributes to more accurate tumor segmentation and classification while aiding clinicians in interpreting the results.

Min-Max Normalization: Normalizing pixel intensities to a range of [0, 1] ensures a consistent input scale for all images, facilitating stable training and faster model convergence. This normalization prevents biases in the model caused by varying intensity ranges, thereby improving accuracy and ensuring that the learned features are meaningful and comparable across the dataset.

Anisotropic Diffusion Filtering: Anisotropic diffusion filtering was applied to enhance regions of uniform intensity while preserving edges and intricate patterns. This technique reduces redundant information, sharpens critical features such as tumor boundaries, and highlights diagnostically significant regions. These enhancements lead to better feature extraction by the model, ultimately improving classification performance and making model outputs more interpretable by emphasizing relevant structures. These preprocessing methods optimize the dataset and significantly enhance the model’s ability to identify and classify gliomas accurately. Furthermore, these techniques improve interpretability by ensuring that the features learned by the model align with clinically meaningful patterns in the MRI data.

## 4 Network architecture

**Figure 3:**
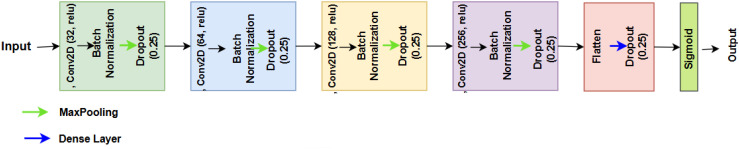
Proposed network.


In this study, we propose a unique CNN architecture as shown in **Figure 3** and described in **Algorithm 1**, for binary classification of brain MRI images, distinguishing between two categories: glioma (tumor) and no tumor. The network receives an image *X* ∈ ℝ*^H^* ^×^ *^W^* ^×^ *^C^*, where *H* and *W* represent the image’s height and width and *C* represents the number of channels (in our example, *C* = 3). The initial layer of the network uses a 2D convolution operation with 32 filters of size 3 × 3 to the input image *X*, succeeded by a ReLU activation function:

$$X_{1}=\text{ReLU}\left(\sum_{i=1}^{32}\left(X\;*\;W_{i}+b_{i}\right)\right)$$

Algorithm 1 **CNN TumorNet.**

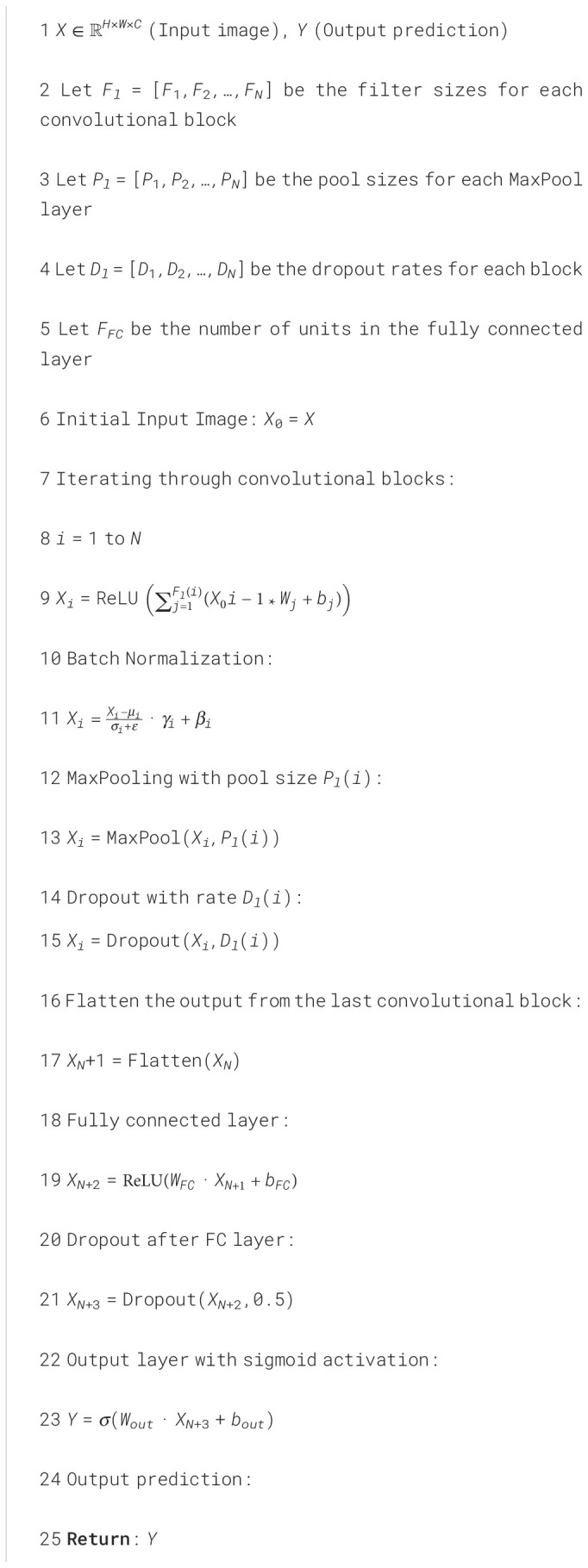

Where *X* ∈ ℝ*^H^* ^×^ *^W^* ^×^ *^C^* is the input MRI, with height *H*, width *W*, and *C* channels. *W_i_* ∈ ℝ^3×3×^ *^C^* are the filter weights for each of the 32 filters (kernel size 3×3, applied across all channels *C*). *b_i_* ∈ ℝ are the bias terms associated with each filter. The operation ∗ represents the 2D convolution between the input image and the filter *W_i_*. The activation function ReLU is applied element-wise after the convolution operation, i.e., ReLU(*x*) = max(0*,x*). We use ‘same’ padding, meaning the resulting dimensions remain identical to the input. The output *X*_1_ is then passed through a batch normalization layer to stabilize the training process:


$$X_{2}=\text{BatchNorm}\left(X_{1}\right).$$

Subsequently, max pooling with a 2 × 2 filter size decreases the spatial dimensions:


$$X_{3}=\text{MaxPool}2\text{D}\left(X_{2},\left(2,2\right)\right).$$

A dropout rate of 0.25 is used for regularization:


$$X_{4}=\text{Dropout}\left(X_{3},0.25\right).$$

The second convolutional block adopts a similar structure, utilizing 64 filters of size 3×3 :


$$X_{5}=\text{ReLU}\left(\sum_{i=1}^{64}\left(X_{4}*W_{i}+b_{i}\right)\right)$$

Where, *X*_4_ ∈ ℝ*^H^*_4_^×^ *^W^*_4_^×^ *^C^*_4_ is the input to this layer, with dimensions *H*_4_, *W*_4_, and *C*_4_ (the output of the previous layer). *W_i_* ∈ ℝ^3×3×^ *^C^*_4_ are the filter weights for each of the 64 filters (kernel size 3 × 3, applied across *C*_4_ channels). *b_i_* ∈ ℝ are the bias terms associated with each filter. The operation ∗ refers to the 2D convolution between the input *X*_4_ and the filter *W_i_*. The activation function ReLU is applied element-wise following the convolution procedure, i.e., ReLU(*x*) = max(0*,x*).


$$X_{6}=\text{BatchNorm}\left(X_{5}\right)$$

Where batch normalization is applied element-wise to normalize the activations of *X*_5_, which helps in accelerating training and stabilizing the learning process. Next, max pooling with a filter size of 2 × 2 is applied to decrease spatial dimensions:


$$X_{7}=\text{MaxPool}2\text{D}\left(X_{6},\left(2,2\right)\right)$$

The max pooling operation captures the maximum value in each 2 × 2 window, effectively reducing the spatial size of the feature map. Finally, dropout with a rate of 0.25 is used to regularize the model and reduce overfitting:


$$X_{8}=\text{Dropout}\left(X_{7},0.25\right)$$

Where dropout randomly sets 25% of the activations to zero during training, preventing the network from relying too heavily on any one neuron. Similarly, we added two additional convolutional layers, followed by batch normalization (BN), max pooling, and dropout layers, using 128 and 256 filters, respectively.


$$X_{9}=\text{ReLU}\left(\sum_{i=1}^{128}\left(X_{8}\;*\;W_{i}+b_{i}\right)\right)$$

Where, *X*_8_ ∈ ℝ*^H^*_8_^×^ *^W^*_8_^×^ *^C^*_8_ is the input to this layer, with dimensions *H*_8_, *W*_8_, and *C*_8_ (the output of the previous layer). *W_i_* ∈ ℝ^3×3×^ *^C^*_8_ are the filter weights for each of the 128 filters (kernel size 3 × 3, applied across *C*_8_ channels).


$$X_{10}=\text{ReLU}\left(\sum_{i=1}^{256}\left(X_{9}*W_{i}+b_{i}\right)\right)$$

Where, *X*_9_ ∈ ℝ*^H^*_9_^×^ *^W^*_9_^×^ *^C^*_9_ is the input to this layer, with dimensions *H*_9_, *W*_9_, and *C*_9_. *W_i_* ∈ ℝ^3×3×^ *^C^*_9_ are the filter weights for each of the 256 filters (kernel size 3 × 3, applied across *C*_9_ channels).

Following the convolutional and pooling layers, the feature map is reshaped into a 1D vector:


$$X_{11}=\text{Flatten}\left(X_{10}\right).$$

This flattened vector *X*_11_ ∈ ℝ*^N^*, where *N* is the number of features, is then passed to a fully connected (dense) layer with 512 neurons and ReLU activation:


$$X_{12}=\text{Dense}(X_{11},512,\text{activation}=ReLU).$$

A dropout layer with a 0.25 rate is introduced to help mitigate overfitting


$$X_{13}=\text{Dropout}\left(X_{12},0.25\right).$$

Lastly, the resultant layer consists of a single neuron with a sigmoid activation function, which produces a probability *y* ∈ [0,1] Indicating the probability that the input MRI image is classified as belonging to the glioma (tumor) class:


$$y=\text{Dense}(X_{13},1,\text{activation}=\text{sigmoid}).$$

The classification decision is determined by the value of *y*: if *y* ≥ 0.5, the model forecasts the image contains a glioma (tumor), and if *y<* 0.5, the image is classified as “no tumor.”

This technique adeptly extracts hierarchical features from input MRI images, systematically diminishing spatial dimensions while encapsulating progressively intricate information. The model guarantees strong performance on novel data by using dropout and batch normalization approaches, effectively mitigating overfitting. The model’s last component, a sigmoid output layer, yields a probability interpreted as the classification outcome for each image. The precise configurations employed in the network, including learning rate, layer count, and dropout rates, are outlined in **Table 1**, highlighting the network’s optimized hyperparameters for enhanced performance. In our study, we selected a dropout rate of 0.25 based on empirical results from initial experimentation. We tested several dropout rates ranging from 0.1 to 0.5 and found that 0.25 provided the best balance between reducing overfitting and maintaining model accuracy. Lower rates did not effectively reduce overfitting, while higher rates led to underfitting. We chose this rate after evaluating model performance on the training and validation sets and observed that this rate helped the model generalize better without compromising its ability to learn the data effectively.

**Table 1:** CNN model hyperparameters.

Hyperparameter	Value
Input Shape	(300,300,3)
Filters	32, 64, 128, 256
Kernel Size	(3, 3)
Activation	ReLU (for Conv2D and Dense layers)
Padding	Same
MaxPooling2D Pool Size	(2, 2)
Dropout Rates	0.25 (Conv2D layers), 0.5 (Dense layer)
Dense Layer Units	512
Output Layer Units	1
Output Layer Activation	Sigmoid

## 5 Explainability

Explainable Artificial Intelligence (XAI) techniques elucidate the rationale behind profound learning model predictions, offering clinicians insights into the foundational logic of a model’s conclusions (19). This is particularly critical in medical environments where the consequences of misdiagnosis are significant, potentially resulting in grave results (20). Incorporating explainability enables AI models to uncover and mitigate biases more effectively, ensuring their adaptability across diverse patient demographics and clinical environments (21). XAI is essential for model validation and regulatory approval, enabling healthcare professionals and regulatory bodies to scrutinize AI decision-making processes, thereby ensuring compliance with ethical and legal standards in clinical applications (7). The advantages of XAI in glioma identification are numerous, encompassing greater diagnostic precision, heightened clinician confidence, and better patient outcomes. XAI improves AI systems by fostering transparency in decision-making and enabling more effective integration of sophisticated computational techniques into clinical practices (22). The integration of deep learning (DL) with explainable AI presents considerable potential for enhancing the detection and treatment of gliomas (23), hence increasing survival rates and improving the quality of life for people afflicted by this severe ailment.

**Figure 4:**
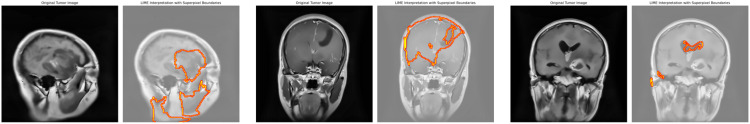
Original image and its corresponding predicted image with LIME highlighting key regions influencing the model’s decision.

LIME (Local Interpretable Model-Agnostic Explanations) is a method in explainable artificial intelligence (XAI) that elucidates individual predictions of any machine learning model by locally approximating its behavior using a more straightforward, more interpretable approach (11). Our research employs LIME to examine the predictions generated by the CNN-TumorNet model, which is intended for brain tumor classification. LIME functions by altering the input data and generating synthetic samples to analyze the impact of modifications on the model’s predictions. This method is crucial for determining the essential characteristics that affect a specific prediction. Our methodology integrates LIME with superpixel segmentation to improve the clarity and depth of the explanations offered. This integration facilitates a more nuanced and thorough comprehension of the elements influencing the model’s judgments. **Figure 4** presents the original MRI image in conjunction with its LIME interpretation, visually depicting the interpretative process and emphasizing the critical regions that influence the model’s classification results.

Superpixel segmentation is a method employed to partition brain images into smaller, coherent units termed superpixels, which encapsulate specific features and enhance detailed image processing. In malignant brain imaging, superpixels facilitate the interpretation of model predictions by emphasizing critical regions typically linked to malignancies or certain glioma traits. Our work identifies three superpixels crucial to the algorithm’s decision-making process for identifying each cancerous image. This identification facilitates a focused analysis of the most critical areas. Integrating the LIME framework enables a precise and localized comprehension of the model’s decision-making by separating specific superpixels. LIME concentrates on aspects the model identifies as significant, such as atypical tissue patterns or tumor margins, offering enhanced clarity regarding the prediction mechanism. This method dramatically improves the system’s transparency and reliability. Emphasizing the most critical superpixels synchronizes the model’s predictions with medical knowledge, identifies areas for enhancement, and guarantees the results are physiologically pertinent. This approach verifies the predictions, facilitates debugging, and enhances the model’s performance. Ultimately, it enhances openness, fosters clinical integration, and bolsters trust in the system’s efficacy in medical diagnostics. While LIME enhances the transparency of our CNN-TumorNet model by highlighting three critical superpixels in glioma MRI images, it has some limitations. LIME uses simpler models like linear regression to explain predictions, which may oversimplify the complex patterns learned by the DL model. Additionally, the perturbations LIME generates to analyze predictions might not always reflect realistic tumor variations, which can lead to less accurate explanations. Focusing only on three superpixels provides localized insights but may miss critical contexts, such as the relationship between the tumor and surrounding regions. Lastly, the identified superpixels might not align perfectly with clinically relevant boundaries based on the model’s learned features rather than anatomical accuracy. These limitations highlight areas for improvement in creating more reliable and clinically meaningful explanations.

## 6 Results

**Figure 5:**
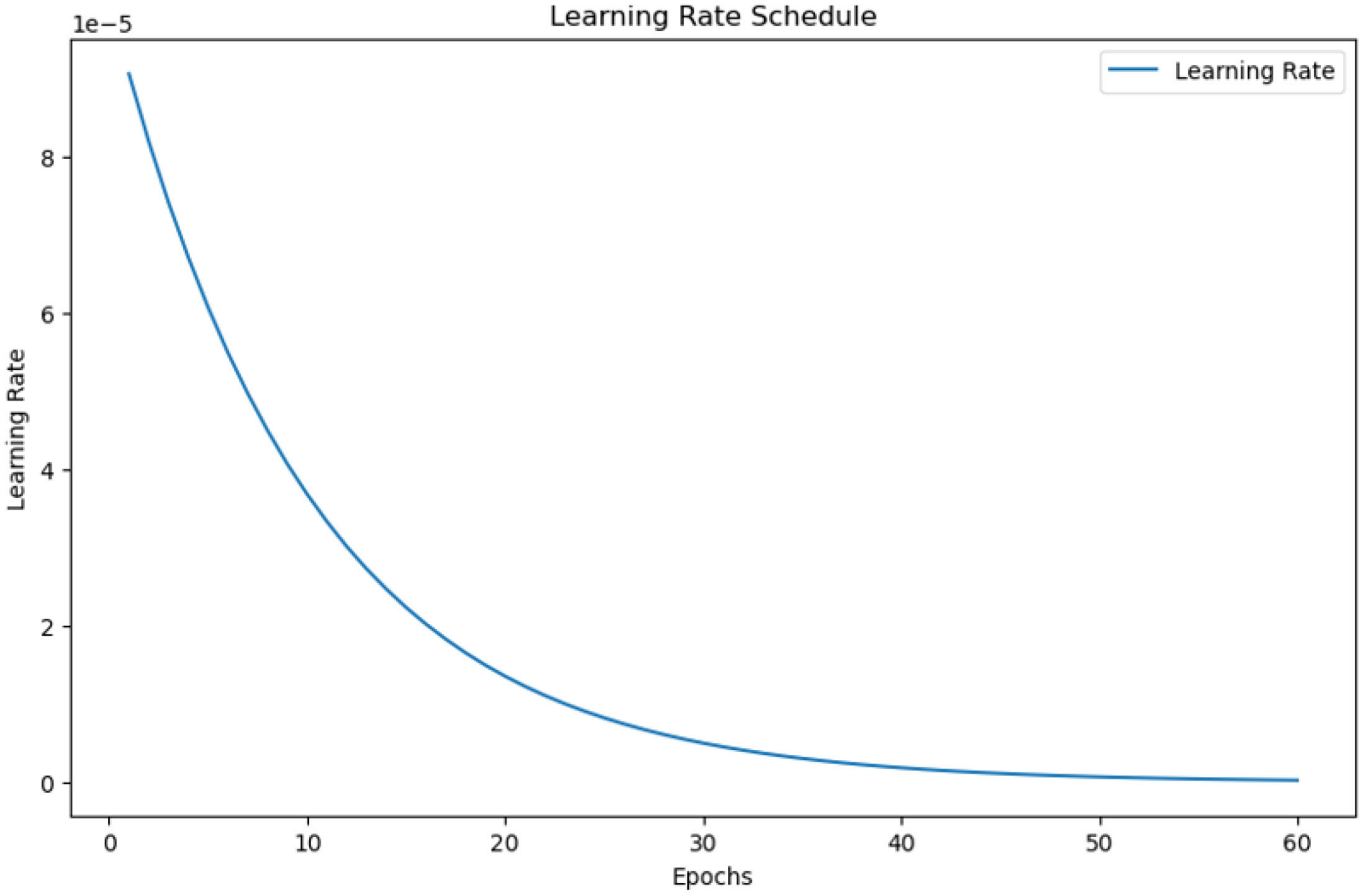
Learning rate progression over epochs during model training.

Initially, the images were of different sizes, so we changed their size to 300x300x3 for consistency. We created data generators for both the training and validation. Furthermore, for data augmentation, we use various methods, such as rotations, shearing, zooming, shifting, flips, and rescaling, to standardize the images in the training set. These augmentations boost model generalization by exposing it to more varied data. Only rescaling was used on the validation set to preserve data integrity and ensure fair evaluation. The MRI images, such as normal and abnormal, were kept in one directory and divided into training and validation sets. The dataset was divided in an 80-20 ratio, with 80% utilized for training and 20% for validation. This division confirms that the model meets a diverse range of images during training, whereas the validation set offers an unbiased assessment of the model’s performance. The 80-20 split reduces overfitting by enabling the model to correctly generalize to previously unknown data during the validation phase. **Figure 5** shows that the model was trained for 60 epochs with the Adam optimizer and a learning rate 0.0001. The binary cross-entropy loss function was chosen as it is suitable for binary classification tasks. We employed an NVIDIA A100 Tensor Core GPU with 80 GB of memory to perform the brain tumor classification experiment. The experiment was implemented using TensorFlow 2.9.1 as the primary deep learning framework. Additional preprocessing and data handling were done using NumPy, Pandas, and SimpleITK libraries, ensuring efficient data preparation and model training. We addressed the class imbalance in the dataset through data augmentation, significantly improving our model’s performance. Techniques such as random rotations, elastic deformations, intensity shifts, and cropping were applied to increase the diversity of tumor classes. These augmentations reduced the model’s bias toward dominant courses, leading to a better balance in learning.

**Figure 6:**
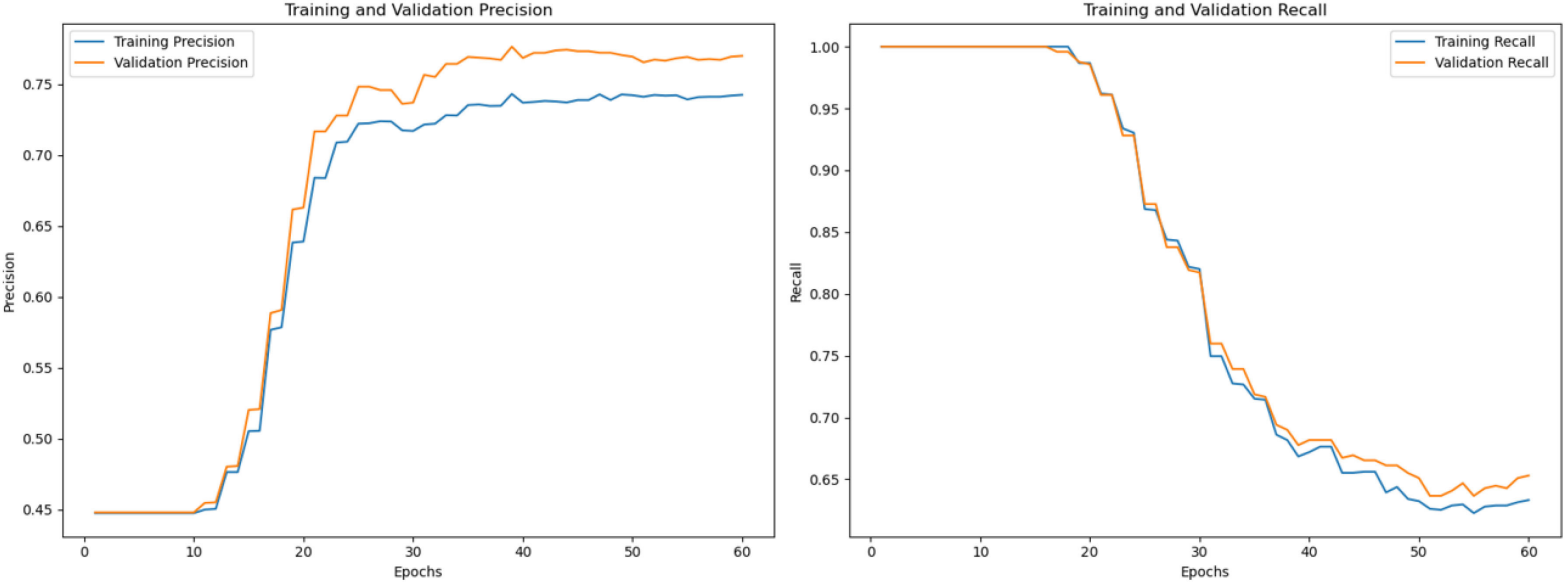
Precision and recall curves for training and validation across epochs.

Next, we load and preprocess the MRI images to ensure they meet the specifications of the CNNTumorNet model. The proposed model achieved outstanding results in the validation phase, as shown in **Figure 6**.

### 6.1 State-of-the-art

**Figure 7:**
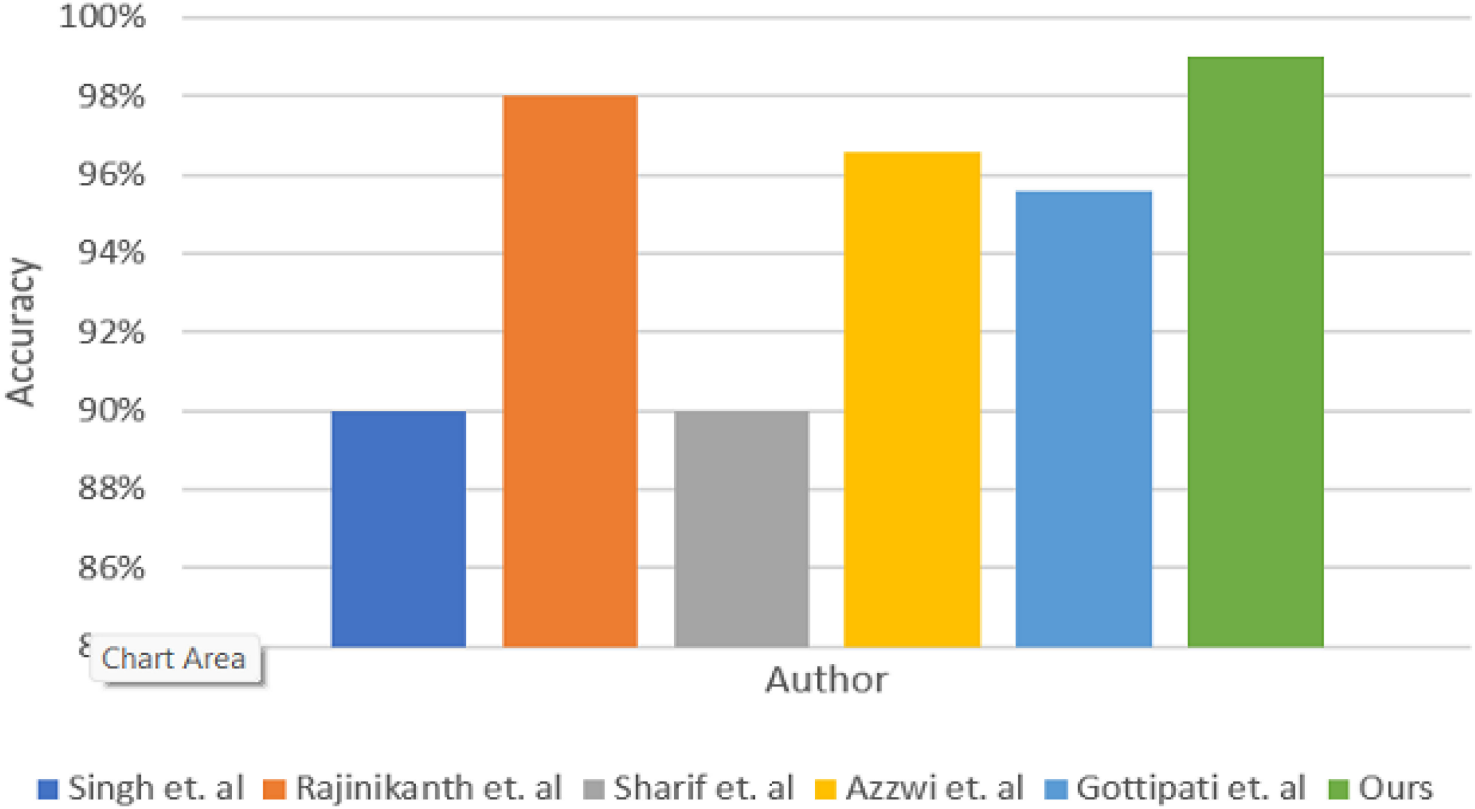
Comparison of CNN-TumorNet with other methods.

Deep learning models have demonstrated exceptional efficacy in brain tumor detection, attaining accuracies beyond 90% through using CNNs, ensemble approaches, and sophisticated optimization techniques, including SVM and genetic algorithms (GA). These findings underscore the possibility of markedly improving diagnostic precision in medical imaging (24) utilized CNN and VGG-16 architectures to classify brain malignancies from MRI images, implementing transfer learning and feature extraction for binary classification (tumor versus no tumor). Their methodology exceeded conventional manual detection techniques employed by clinical experts, with a remarkable 90% accuracy on the test set and 86% on the validation set. Rajinikanth et al. (2021) created a computer-aided disease diagnostic (CADD) system for the detection of brain cancers from MRI images. This system employed CNN-based segmentation and classification, integrating feature extraction and selection in its binary classification procedure. The researchers achieved over 98% accuracy in tumor identification via SVM-Cubic and 10-fold cross-validation, surpassing prior methodologies. Furthermore, a study conducted by (25) presented a strategy for identifying brain cancers in MRI images that enhanced current saliency segmentation and feature selection methodologies. This approach encompassed tumor preprocessing, improved thresholding for segmentation, and SVM classification. A genetic algorithm (GA) was employed to optimize attribute selection, improving the process and augmenting diagnostic performance. These examples highlight the formidable possibilities of DL technology in the accurate and efficient diagnosis of brain cancers. After evaluation, authors obtained over 90% accuracy (26). enhances brain tumor classification by utilizing a stacked ensemble DL framework, incorporating VGG19, Inception v3, and ResNet 10. The model obtained 96.6% accuracy in binary classification (normal vs. aberrant brains) on a Kaggle dataset. It finds that the ensemble method outperforms individual models in tumor prediction (27). proposes a brain tumor classification approach that uses CLBP and CNN to achieve 95.6% accuracy on MRI images. It integrates texture feature extraction and CNN classification to boost diagnostic accuracy. **Table 2** compares the proposed network to existing approaches, and a visual depiction is provided in **Figure 7**.

**Table 2:** Comparison of different methods for brain tumor classification.

Author	Method	Dataset	Accuracy
(24)	CNN and VGG-16	ImageNet Datadase	90%
(28)	SVM-Cubic	GBM/LGG	98%
(25)	SVM	Clinical/Harward dataset	90%
(26)	Stacked Ensemble	Kaggle MRI dataset	96.6%
(27)	CNN	MRI dataset	95.6%
Ours	CNN-TumorNet	Kaggle MRI dataset	99%

## 7 Conclusion and future scope

This paper presents CNN-TumorNet, a sophisticated and resilient approach for categorizing brain tumors in MRI images, differentiating between tumor and non-tumor conditions. Our model exhibited remarkable performance on the Kaggle MRI dataset, indicating a substantial accuracy level and highlighting its potential for early tumor detection in medical imaging. Our research primarily addresses the interpretability difficulties frequently encountered with DL models. Although deep neural networks excel in numerous domains, their black-box nature poses a significant obstacle in key areas like healthcare, where comprehending the decision-making process is vital. To address this, we incorporated the LIME technique into our framework. LIME improves our model’s transparency by clarifying the specific regions of MRI images, which significantly impacts the categorization decisions for malignant gliomas. This enhanced interpretability augments the reliability and credibility of CNN-TumorNet’s predictions and strengthens its adoption within the healthcare sector. Moreover, using explainability tools such as LIME enhances user confidence in our AI system, rendering it a more ethical and pragmatic choice for real-world medical applications. Looking ahead, this study lays the foundation for advancing AI-driven medical image processing, with significant implications for future research and healthcare applications, particularly in neurology. Refining explainability techniques remains a key area for development, as it could provide deeper insights into model decision-making and foster trust among clinicians. Expanding the model to categorize a broader range of brain tumor types and neurological conditions could significantly enhance its utility in clinical practice. Additionally, improving the model’s computational efficiency and robustness would facilitate its integration into automated image processing pipelines, enabling real-time applications in diverse healthcare environments. These advancements can potentially revolutionize brain disorders’ detection, diagnosis, and treatment, driving innovation in AI’s role within neurology and beyond.

## 8 Limitations of the current study

The CNN-TumorNet model demonstrates remarkable accuracy in brain tumor segmentation from MRI images and utilizes the LIME technique to improve interpretability, guaranteeing that model predictions are comprehensible and transparent. This methodology validates the model’s applicability in clinical environments; nevertheless, additional verification with a broader range of clinical datasets would enhance its reliability and efficacy across various imaging protocols and institutional procedures. Moreover, although LIME is crucial for elucidating the model’s decision-making for specific instances, broadening the interpretability to yield more extensive, global insights could enhance clinicians’ comprehension of the model’s predictions across diverse brain tumor types and patient conditions. Improving these facets of the model should enable more seamless integration into various clinical workflows, ensuring it adjusts effectively to differing conditions without incurring substantial computing requirements. This comprehensive strategy would facilitate the model’s preparedness for widespread implementation and practical application across various medical environments.

## Data Availability

The original contributions presented in the study are included in the article/supplementary material. Further inquiries can be directed to the corresponding author.
